# Applying the Stimulus - Organism - Response framework for exploring use intention - a case study for a digital cultural and creative product, The *Forbidden City 365* app

**DOI:** 10.1371/journal.pone.0343931

**Published:** 2026-03-09

**Authors:** Xin Pan, Mingming Zhong

**Affiliations:** 1 School of Fine Arts, Guangdong University of Education, Guangzhou, Guangdong Province, China; 2 School of Fine Arts, Tsinghua University, Beijing, China; Macao Polytechnic University, MACAO

## Abstract

At present, little is known about the behavioral mechanisms underlying “individuals” use of digital cultural and creative products (DCCPs). To fill this research gap, this study investigates the key determinants influencing users’ use intention, with a particular focus on how external stimuli affect users’ behavioral intentions through their psychological and emotional states. This study adopts the Stimulus–Organism–Response (SOR) model and takes the *Forbidden City 365* app as a case to explore the key experiential factors influencing users’ use intention of digital cultural and creative products, using Structural Equation Modeling (SEM) as the analytical method. Data were collected through a questionnaire survey, yielding 403 valid responses. Media richness significantly enhances users’ perceived cultural value and satisfaction; design aesthetics exerts a significant positive impact on perceived cultural value, satisfaction, and cultural identity; and high culture effectively strengthens satisfaction and cultural identity. Meanwhile, perceived cultural value, satisfaction, and cultural identity, as key mediating mechanisms, all exert significant positive predictive effects on users’ use intention. The findings support the proposed theoretical hypotheses. They suggest that the development of DCCPs should emphasize in-depth cultural expression, diversified media presentation, and optimized aesthetic design. Such an approach can achieve the dual goals of effective cultural communication and enhanced user experience.

## 1. Introduction

As the digital economy has thrived in recent years, the digital creative industry has experienced rapid growth [1]. Centered on cultural heritage resources, this industry integrates digital technologies, creative design, and industrialization pathways. It aims to promote the digital development, innovative expression, and market transformation of cultural resources. In doing so, it effectively contributes to the preservation, dissemination, and revitalization of traditional culture [2,3]. This trend not only reflects the practical demand for the integration of culture and technology, but also demonstrates the public’s multidimensional aspirations for cultural inheritance, aesthetic experience, and spiritual value in the context of a new era. Against this backdrop, digital cultural and creative products (DCCPs) taken on a central role in fostering cultural innovation [4,5].

DCCPs are defined as a new form of creative design that utilizes digital technology as a medium to integrate cultural elements, creativity, and product attributes in an organic way, conveying cultural connotations through the integration of virtual and physical experiences [6,7]. The core characteristics of DCCPs lie in the combination of cultural value, creative design, and digital presentation. They encompass not only digitalized cultural exhibits, virtual cultural and creative products, and digital artworks, but also cultural experiences and services delivered through digital platforms [8]. Existing studies have mainly focused on the development technologies, user experiences, and cultural communication effects of digital cultural products, while research on user interaction mechanisms, usage intentions, and psychological influences remains relatively limited.

DCCPs not only enrich users’ modes of cultural experience [9] and expand the channels through which culture is accessed [10] but also stimulate users’ proactive engagement in exploring, understanding, and re-creating cultural content [11]. Moreover, these products bridge online and offline dissemination channels, extending the pathways for cultural contact and participation [12], thereby facilitating the shift of culture from “passive reception” to “active participation” [4]. In addition, DCCPs are increasingly becoming an emerging engine powering digital economic growth. Cultural creativity breaks through the rigid patterns of traditional product design and production, enhancing the market value and competitiveness of cultural products [13].

However, some scholars argue that although DCCPs demonstrate significant potential in activating cultural resources, expanding cultural dissemination pathways, and driving digital economic development, they still face multiple challenges during their development. At present, many products lack depth in aesthetic value presentation [14], exhibit a low degree of integration with digital technologies [4,15], and convey cultural connotations superficially [16], making it difficult to achieve profound transmission of cultural meaning. These issues to some extent hinder users’ willingness to use DCCPs [14]. Therefore, it is necessary to more deeply explore the determinants that affect and sustaining users’ use intention toward DCCPs, which has been insufficiently addressed in earlier research.

To address the lack of research on the behavioral mechanisms of users of DCCPs, this research investigates the main determinants of user engagement, with a particular focus on how external stimuli affect users’ behavioral intentions through their psychological and emotional states. To this end, Stimulus–Organism–Response (SOR) model [17] as its theoretical foundation. The SOR framework posits that external stimuli (Stimulus) influence individuals’ internal states (Organism), which in turn elicit corresponding behavioral responses (Response). Against the backdrop of DCCPs, this study conceptualizes (S1) media richness, (S2) design aesthetics, and (S3) high culture content as external stimulus variables, and investigates how these factors affect users’ emotional and cognitive responses—namely, (C1) perceived cultural value, (C2) satisfaction, and (C3) cultural identity—which subsequently shape their behavioral response, specifically (U1) use intention. Based on this theoretical foundation, the study addresses two core research questions (RQs) aimed at exploring how design features impact user behavior through internal psychological mechanisms, thereby offering theoretical guidance for the optimized design and strategic dissemination of DCCPs.

RQ1: In the context of DCCPs which stimulus factors (e.g., media richness, design aesthetics, and cultural content) significantly influence users’ psychological and emotional states?

RQ2: Under the SOR framework, how do users’ psychological and emotional states function as mediating mechanisms to further affect their behavioral use intention toward DCCPs?

Furthermore, by investigating the mechanisms through which the characteristics of DCCPs impact user behavioral intentions, the findings enrich current literature across pertinent fields of study. It clarifies how designers can more effectively utilize design elements to enhance user experience and support the development of the digital cultural and creative industry. At the same time, it offers valuable practical implications for cultural and creative organizations seeking to stimulate user interest and improve the effectiveness of cultural dissemination.

The structure of the paper’s structure is presented as follows. First, A review of the pertinent literature is conducted, followed by the formulation of research hypotheses. Second, this section outlines the quantitative methods utilized in the present research. Third, this part reports and explains the outcomes generated from the survey data and SEM analysis. This is followed by a discussion of the theoretical and practical implications of the findings. The conclusion summarizes the primary outcomes, discusses their scholarly and real-world importance, and puts forward the study’s shortcomings and recommendations for subsequent research.

## 2. Literature review and hypothesis development

### 2.1. Digital Cultural and Creative Products (DCCPs)

Cultural authorities are increasingly recognizing the strategic significance of digital technologies in advancing the safeguarding and creative revitalization of cultural heritage. These technologies are not only seen as efficient media for cultural dissemination, but also play a crucial role in the protection and revitalization of cultural heritage [18–20]. DCCPs, rooted in cultural content, achieve information exchange between users and culture through the in-depth exploration of cultural symbols and meanings, as well as the creative interpretation and representation of cultural elements. These products exist in forms that are both material and immaterial, enabling multi-dimensional engagement [6].

DCCPs are increasingly diversifying in both their expressive forms and functional connotations. For example, they utilize digital technologies to enable bidirectional interaction among people, cultural artifacts, and data, thereby enhancing users’ sense of immersion and cultural participation [21]. Digital media technologies provide a novel narrative context for cultural content, facilitating the development of video-based cultural products and strengthening the visual expression and communicative power of cultural information [22].

### 2.2. Stimulus–Organism–Response (SOR) Model

Mehrabian and Russell [23] first proposed the Stimulus-Organism-Response (SOR) model. According to this model, environmental stimuli can influence users’ cognitive and emotional states. It posits that environmental cues act as external stimuli (S), which affect the internal cognitive and emotional states of the organism (O), thereby leading to behavioral responses (R).

Originally developed as a cognitive framework in psychology, the SOR model effectively reflects internal psychological processes, making it particularly suitable for capturing the complexity of users’ psychological experiences [24]. In recent years, scholars have widely applied the SOR framework in digital environment research [25–27]. In such contexts, external stimuli are often closely associated with characteristics of the digital environment, such as aesthetics [28], modes of interaction [29], multimedia presentation, information quality, system quality, and service quality [27]. Such features influence both thought patterns and emotional states, which consequently inform users’ behavioral tendencies.

In this study, the design elements of DCCPs are regarded as external stimulus factors. The “organism” component of the SOR model represents users’ internal states and serves as a critical mediating variable, encompassing personal experiences, perceptions, and emotional responses [30,31]. Accordingly, this study treats certain core perceptions in the user experience as organism variables to capture users’ psychological responses during their engagement with DCCPs. Ultimately, use intention reflects a combined response to both external stimuli and internal states, representing the “response” dimension of the SOR model.

### 2.3. Media richness

Media richness, proposed by Daft and Lengel [32], refers to a medium’s capacity to convey information within a specific time frame. The effectiveness of information transmission depends on a medium’s ability to deliver feedback, nonverbal cues, and linguistic variety. The richer the medium, the more accurately and efficiently users can comprehend the conveyed information. In digital environments, multimedia functions—such as the integration of images, audio, video, and interactive features—significantly enhance the expressive power of media, thereby improving users’ reception and understanding of information. Especially when dealing with ambiguous or unstructured tasks, high media richness can effectively reduce misunderstandings and improve communication efficiency [33]. Media richness has been shown to significantly influence user behavior across various digital contexts, including instant messaging platforms [34], e-commerce websites [35], digital marketing channels [36], and user intention in digital museums [37]. These studies consistently demonstrate that greater media richness enhances the effectiveness of information transmission and increases users’ comprehension and acceptance of media content.

According to media richness theory, higher levels of media richness can improve the clarity of information and users’ understanding, thereby influencing their behavioral responses [32]. Lin et al. [38] further pointed out that in the context of online purchases of cultural and creative products, high media richness plays a significantly positive role. Diverse media presentation formats can more comprehensively showcase the essence of cultural and creative products, enhance users’ perceived attractiveness and anticipation, and thus improve their perceived cultural value and satisfaction. Therefore, this study hypothesizes that using multimedia technologies—including images, music, videos, and interactive features—to present traditional cultural resources such as the Forbidden City in a multidimensional way can stimulate user interest and cultural expectations from multiple perspectives, thereby significantly enhancing their satisfaction and perceived cultural value toward DCCPs.

H1: Media richness has positive effects on perceived cultural value.

H2: Media richness has positive effects on satisfaction.

### 2.4. Design aesthetics

The term “aesthetics” originates from the Greek word *aisthetikos*, which pertains to sensory perception. Design aesthetics represents a holistic cognitive perspective, wherein the construction and perception of any object involve specific design elements (e.g., shape, color, light and shadow) and design principles (e.g., unity, contrast, balance, and proportion) [39]. Toufani et al. [40] described design aesthetics as the sensory perception of beauty, order, and harmony. In the context of digital design, design aesthetics is commonly defined as the visual appeal of a design and its capacity to convey a clear and unique image [41]. Visual attractiveness is manifested through the use of elements such as color, typography, graphics, and imagery, which collectively influence users’ perception and emotional experience.

As digital products increasingly serve as platforms for user interaction and cultural expression, they have gradually come to be regarded as aesthetic objects themselves. Research indicates that most users tend to evaluate and select products from an aesthetic perspective [14], making aesthetics an indispensable component of user experience [42]. Existing studies show that design aesthetics is not only a positive product attribute that can enhance a product’s perceived value and influence consumer preferences [43], but also improves user satisfaction and intention to use [44,45]. When DCCPs skillfully integrate traditional cultural symbols and aesthetic elements in their design, they can evoke users’ emotional resonance with familiar cultures and thereby enhance cultural identity [46]. Cultural and creative products with higher levels of design aesthetics are more likely than those with lower aesthetic appeal to stimulate users’ perception of the product’s cultural connotations and symbolic value. As Schultz [47] pointed out, design aesthetics has a significant positive impact on consumers’ perceived value. A high-quality visual and aesthetic experience not only increases users’ identification with the product but also strengthens their understanding and recognition of the cultural value embodied by the product. Therefore, the effective application of design aesthetics in the design process of DCCPs is of critical importance for enhancing users’ perceived value, satisfaction, and cultural value. Consequently, this research sets out the following hypotheses:

H3: Design aesthetics has positive effects on cultural identity.

H4: Design aesthetics has positive effects on satisfaction.

H5: Design aesthetics has positive effects on perceived cultural value.

### 2.5. High culture

Gans [48] categorized culture into two main types: “high culture” and “popular Culture.” High culture primarily includes classical music, literary classics, fine arts, theater, and opera, which are typically characterized by high complexity, intellectual depth, and historical significance. In contrast, popular culture emphasizes entertainment and accessibility, targeting a broader consumer base. High culture not only reflects the intrinsic artistic value of works but also represents the aesthetic taste and value system of cultural elites, serving as an important vehicle for the expression of the ideas and emotions of the “cultured” class.

Li [49] defines high culture as cultural forms possessing a high level of artistic quality and aesthetic value. As a vital component of cultural experience, high culture holds a significant position in traditional Chinese culture [50,51]. High culture contents, such as traditional architecture, classical literature, traditional arts, and music, not only carry profound cultural heritage but are often regarded as symbols of social identity and cultural literacy. Individual identification with such cultural heritage helps reinforce cultural belonging and self-identity, thereby promoting cultural transmission and preservation [52].

In cultural experience activities, integrating high culture elements contributes to enhancing the cultural refinement and aesthetic value of products, as well as increasing user satisfaction [49]. Studies have shown that users with a strong orientation toward high culture tend to have higher frequency and deeper engagement with digital media, especially favoring digital products with artistic and cultural depth [53]. These users are more likely to derive aesthetic pleasure and psychological fulfillment when interacting with DCCPs that incorporate high culture content, resulting in profound value recognition and emotional resonance. The effective integration of high culture elements not only improves overall user experience but also plays a positive role in fostering cultural awareness and strengthening intentions for cultural preservation. Drawing on the preceding analysis, the study puts forward the following hypotheses:

H6: High culture has positive effects on satisfaction.

H7: High culture has positive effects on cultural identity.

### 2.6. Perceived cultural value

The concept of “perceived value” was first introduced by Porter [54] in Competitive Advantage and has since been extensively developed within consumer behavior research. According to Zeithaml [55] perceived value refers to a consumer’s overall judgment regarding a product or service, based on perceptions of what is received and what is given. Eggert and Ulaga [56] further emphasized that consumers tend to choose products that maximize their perceived value, highlighting perceived value as a key driver of user behavior. This theory has gradually been applied across multiple fields, including cultural consumption, digital media, and creative design [46], becoming an important variable for measuring user experience and behavioral intention.

Perceived cultural value is regarded as an extension of perceived value within the domain of cultural consumption. Cultural value emphasizes the spiritual connotations, historical heritage, aesthetic features, and identity-related symbolic meanings embedded in cultural products [57]. As Pierre Bourdieu’s seminal work Distinction: A Social Critique of the Judgement of Taste [58] illustrates, society operates within a multidimensional space defined by economic capital (wealth) and cultural capital (education and taste). Taste functions as a “system of classification” that not only categorizes objects (e.g., preferring Mozart over popular music) but also classifies people, drawing symbolic boundaries between social groups. Cultural producers employ their artistic “distinctiveness” to differentiate themselves from economically dominant classes and mass culture, highlighting the social function of aesthetic preference. Therefore, unlike traditional value assessment systems centered on economic returns, cultural value focuses more on its role in social recognition, cultural transmission, and emotional connection [59]. Accordingly, perceived cultural value is defined as users’ subjective cognition and emotional engagement with the cultural meanings, aesthetic characteristics, and symbolic significance embodied in cultural products during the consumption process [60]. This construct reflects a deep understanding and emotional participation of users toward cultural products and serves as an important psychological mechanism by which cultural products influence user behavior.

As a critical mediating variable between cultural experience and user behavior, perceived cultural value has been confirmed to play a central role in DCCP design and user research [46,61]. When users perceive the historical, aesthetic, and spiritual values embedded in digital cultural creative products, they have a higher probability of positive cognitive evaluations and emotionally resonate with the culture. This perceived cultural value not only enhances users’ overall satisfaction with the product but also significantly increases their use intention [62]. Therefore, by enriching cultural connotations and enhancing users’ perception of cultural experience, DCCPs can more effectively attract users and promote their use intentions.

H8: Perceived cultural value has positive effects on use intention.

### 2.7. Satisfaction

Satisfaction has long been regarded as one of the core variables in the fields of marketing and behavioral science, serving as an important indicator for predicting users’ attitudes and behaviors. As early as 1965, Cardozo introduced satisfaction into marketing research and pointed out that customer satisfaction significantly influences future use intentions [63]. Within the SOR framework, satisfaction is typically viewed as a subjective response and psychological state formed after the use of a product or service, reflecting the individual’s overall evaluation of the experience outcome [64]. When a product or service meets users’ needs, expectations, or emotional appeals, users are more likely to develop a higher level of satisfaction, which in turn leads to more positive behavioral responses. For example, Kurnaz et al. [65] found in the context of digital that satisfaction has a significant positive impact on users’ use intentions and is one of the key drivers in their behavioral decision-making process.

In this study, satisfaction is used to assess and explain users’ use intentions toward DCCPs. Taking the *Forbidden City 365* as an example, when users are satisfied with their experience using the product, they are more likely to continue engaging with the platform. Conversely, if the experience is unsatisfactory, users may discontinue use and turn to competing products. Therefore, the following hypothesis is proposed:

H9: User satisfaction has positive effects on use Intention.

### 2.8. Cultural identity

From an individual perspective, Erikson [66] conceptualized cultural identity as a process through which individuals internalize the culture of their group, thereby generating a sense of belonging. This process reflects the socio-psychological mechanisms by which individuals acquire, maintain, and innovate their cultural identity within society. From a broader viewpoint, Phinney [67,68] defined cultural identity as an individual’s emotional attachment and mental affiliation with a specific ethnic or national group—a clear and assured acceptance of one’s own culture. Similarly, Jensen et al. [69] emphasized that cultural identity involves the sense of belonging individuals or groups experience within their cultural context. This identity not only manifests as cultural acceptance and confidence but also becomes deeply internalized as self-awareness, enhancing cultural confidence and psychological adaptation [70].

Cultural identity plays a key role in user experience and behavioral decision-making. On one hand, it helps cultivate a sense of belonging and enriches user experiences [71,72]. On the other hand, users often express their cultural affiliation through cultural heritage and related products [73], and their usage decisions are significantly influenced by cultural identity [74]. Prior research indicates that cultural identity has a direct and significant positive impact on users’ perceived emotional value [75], which in turn stimulates purchase intention [76]. As a vital component of users’ internal psychological mechanisms, cultural identity should be considered a core factor in the design strategies of DCCPs. The following hypothesis is proposed:

H10: Cultural identity has positive effects on use Intention.

### 2.9. Mediating hypotheses

When users encounter DCCPs with cultural depth and aesthetic appeal, they perceive the cultural value embedded in the products [77,78]. This perception enhances their emotional experiences, such as interest and pleasure [79]. It also motivates further cultural participation and willingness to share. During cultural experiences, users’ positive emotional responses—such as enjoyment and immersion—strengthen their overall satisfaction. Satisfaction serves as a crucial link between psychological experiences and behavioral reactions, including repeated use and recommendation [80]. DCCPs are not only aesthetic and interactive media but also carriers of cultural identity. When users resonate emotionally with the products, they reinforce their sense of cultural identity and belonging. This strengthened cultural identity further drives their willingness to share, promote, and continuously engage with DCCPs [63].

This study posits that users’ internal psychological and emotional states—namely perceived cultural value, satisfaction, and cultural identity—mediate the effects of DCCP stimuli (media richness, design aesthetics, and high culture) on users’ behavioral use intention. Accordingly, the following hypotheses are proposed:

H11: Users’ perceived cultural value mediates the relationships between DCCP stimuli (media richness, design aesthetics, and high culture) and their use intention.

H12: Users’ satisfaction mediates the relationships between DCCP stimuli and their use intention.

H13: Users’ cultural identity mediates the relationships between DCCP stimuli and their use intention.

**Fig 1:**
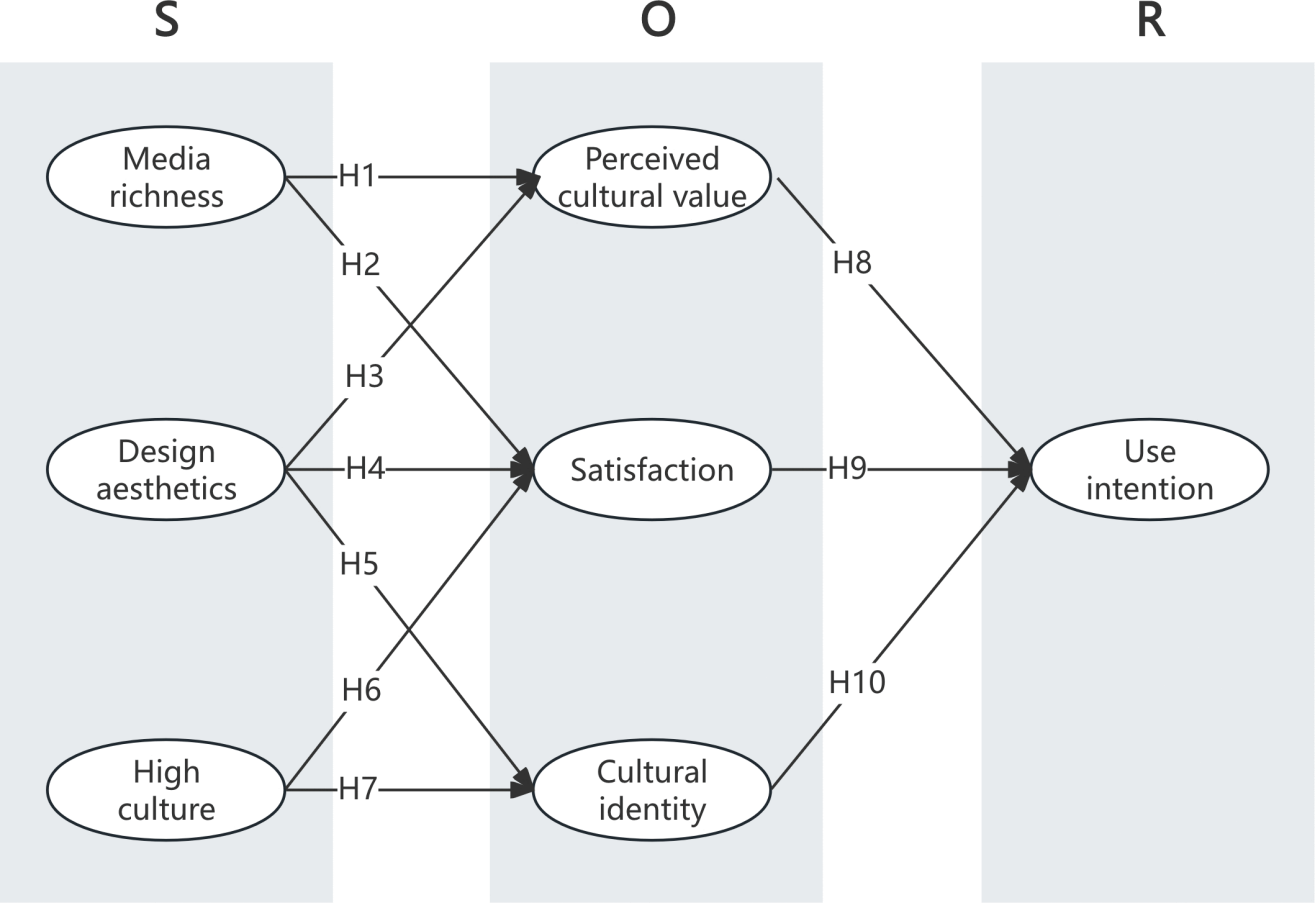
The research model.

The proposed research model of this study is shown in Fig 1.

## 3. Methodology

### 3.1. Background —— the *Forbidden City 365* app

Developed by the Palace Museum, the *Forbidden City 365* application is a representative example of DCCPs. The app enables users to interact with the cultural heritage of the Forbidden City through various interactive features. It offers more than 30 thematic levels and over 850 knowledge-based quizzes related to ancient architecture. Users receive achievement badges as rewards for completing these quizzes. In addition, its panoramic mode allows users to explore the main and rear courtyards of the Forbidden City in an immersive way. As a result, the application not only enhances public engagement with cultural heritage but also serves as an important digital platform for preserving and disseminating the architectural craftsmanship of the Ming and Qing dynasties. Therefore, it represents a valuable and representative case for examining user interaction with DCCPs.

### 3.2. Sample

This study is an exploratory investigation aimed at examining users’ intentions to use DCCPs. The target participants were individuals with prior experience using such products. This study adopted a convenient sampling method. An online survey link, accompanied by a brief introduction to the research purpose, was distributed to users across China via the *Wenjuanxing* platform. Data collection was conducted in April 2025. Participants were required to download and use the *Forbidden City 365* mobile application before completing the questionnaire. A screening question was included to verify usage experience, asking participants to confirm whether they had previously used the app. Only responses from participants who confirmed having used the *Forbidden City 365* were considered valid samples. Those who indicated they had not used the app were automatically directed to terminate the survey, and their responses were classified as invalid. To ensure data quality, responses with abnormally short completion times or from participants who had not actually used the app were excluded from the final dataset. After data cleaning, a total of 403 valid responses were retained for analysis.

### 3.3. Ethics statement

The research was conducted using a questionnaire survey, with a total of 403 participants (N = 403) completing the questionnaire. The first page of the questionnaire provided detailed information about the study’s background, objectives, and relevant content. By completing the questionnaire, participants were considered to have read, understood, and voluntarily agreed to participate in the study. In addition, written informed consent was obtained from all participants prior to data collection.

The survey was conducted anonymously, and respondents were free to withdraw at any time. The sampling process ensured that the collected data represented users with genuine experiences of digital cultural interaction. No identifying information about any participant will be disclosed in any report or publication resulting from this research.

All procedures performed in this study were in accordance with the ethical standards of the institutional and national research committees, as well as with the 1975 Declaration of Helsinki (revised in 2000). The research project was reviewed and approved by the Academic Committee of Guangdong University of Education and complied with all relevant ethical standards.

### 3.4. Instrument

The measurement scale in this study focuses on the usage of the *Forbidden City 365* DCCP, aiming to investigate users’ behavior in engaging with DCCPs. The scale is divided into two parts (Table 1): The initial section collects demographic information, including their gender, age, and occupation, as well as a usage screening item, where participants are required to confirm whether they have used the Forbidden City 365 product. The second section addresses the core constructs of this study, each evaluated using a 7-point Likert scale. Participants rated their agreement with each item according to personal experience, with responses ranging from 1 (“strongly disagree”) to 7 (“strongly agree”).

**Table 1:** Measurement items.

Variable	Revised items	Source
Media richness (MR)	MR1: The *Forbidden City 365* can convey information in various ways. MR2: The *Forbidden City 365* conveys content using multiple forms of presentation. (e.g., images, text, videos, animation, audio, and 3D virtual environments). MR3: The *Forbidden City 365* delivers immediate feedback based on my requests. MR4: On the whole, the *Forbidden City 365* provides me with a wealth of information.	[37,38]
Design aesthetics (DA)	DA1: I experienced a strong sense of harmony while engaging with the *Forbidden City 365*. DA2: I believe the *Forbidden City 365* looks very aesthetic. DA3: The *Forbidden City 365* appears to be professionally designed	[46,81]
High culture (HC)	HC1: The high culture of the *Forbidden City 365* with its beautiful images, history, and panoramic architecture is an important part of DCCPs. HC2: I have a deep appreciation for high culture of the Forbidden City such as its architecture and history. HC3: The high culture of the Forbidden City’s history, culture, and architecture can enhance my digital experience. HC4: After this digital experience, I would like to continue to learn about high cultures such as the Forbidden City’s history, culture, and architecture.	[49,51]
Perceived cultural value (PCV)	PCV1: I believe that the *Forbidden City 365* is highly valuable. PCV2: Using the *Forbidden City 365* was a good decision. PCV3: Considering the time I invested, experiencing the *Forbidden City 365* was worthwhile. PCV4: After using the *Forbidden City 365*, my appreciation for Chinese culture deepened on an emotional level.	[59,82]
Satisfa- ction (SAT)	SAT1: My experience with the *Forbidden City 365* was highly satisfying. SAT2: The experience of using the *Forbidden City 365* met my expectations. SAT3: I find the performance of the *Forbidden City 365* to be highly satisfactory. SAT4: Choosing to use the *Forbidden City 365* proved to be a smart decision on my part.	[46]
Cultural identity (CI)	CI1: I have learned more about the culture than I knew before by using the *Forbidden City 365*. CI2: The *Forbidden City 365* has fostered a strong sense of cultural confidence and national pride. CI3: If given the opportunity, I would prefer to spend more time engaging with DCCPs such as the *Forbidden City 365*. CI4: I believe that the *Forbidden City 365* have unique connotations. CI5: I can resonate emotionally when I use the *Forbidden City 365*. CI6: I think the *Forbidden City 365* has effectively promoted the development of intangible cultural heritage.	[72]
Use Intention (UI)	UI1: I have a stronger intention to using the *Forbidden City 365* in the future. UI2: I intend to maintain my use of the *Forbidden City 365* and gradually increase how often I use it. UI3: I highly encourage others to use the *Forbidden City 365*.	[46]

To ensure measurement validity, this study adopted multiple indicators to assess each proposed construct. Wherever possible, well-established and validated scales were used. Given the nature of the research and the characteristics of the respondents, the questionnaire was based on validated scales from similar studies. It was then localized and contextually adapted to the Chinese cultural and linguistic environment. For example, the measurement items for media richness were adapted from two studies on digital cultural and creative products. These adaptations ensured that the items better fit the cultural and digital interaction context explored in this research. The measurement of design aesthetics was also adapted from two studies on behavioral intentions toward cultural and creative products, with modifications for contextual relevance. Generic terms such as “the product” or “this interface” were replaced with the specific name the *Forbidden City 365* to enhance respondents’ immersion and comprehension. To ensure scientific rigor and applicability, a small-scale pilot test was conducted. The results indicated that the questionnaire demonstrated good operability and clarity, effectively capturing the core characteristics of the studied variables.

### 3.5. Data analysis

This study employed SEM to systematically analyze the collected data. SEM, widely used in disciplines such as social sciences, management, and design, is a powerful quantitative research method known for its robust capabilities in model construction and hypothesis testing. It effectively addresses complex relationships among multiple latent variables and their observed indicators [83]. A key advantage of SEM lies in its ability to simultaneously assess the overall model fit and examine both direct and indirect effects among variables, thereby enhancing the scientific rigor, reliability, and external validity of the research findings.

Specifically, data processing and path analysis were conducted using SPSS and AMOS. This research adopted the two-step method outlined by Anderson and Gerbing [83,84]. The first step involved Confirmatory Factor Analysis (CFA) to test the structural validity and reliability of the measurement model, ensuring that the measurement instruments exhibited satisfactory discriminant and convergent validity. The second step involved SEM to evaluate the hypothesized paths among variables, assess model fit indices, and determine the statistical significance of the path coefficients, thereby revealing the causal relationships among the constructs.

## 4. Results

### 4.1. Sample description

The valid sample consisted of 403 participants, including 227 women (56.33%) and 176 men (43.67%). In terms of age distribution, 68 participants (16.87%) were aged 18–25, 196 participants (48.64%) were aged 26–34, 114 participants (28.29%) were aged 35–50, and 25 participants (6.2%) were aged over 50. Regarding educational background, 22 participants had an education level below high school, 327 held a bachelor’s degree, and 54 had a master’s degree or higher. In terms of occupation, 58 participants were students, 134 were corporate employees, 68 were government workers, 64 were professionals in technical fields, 66 were freelancers, 5 were retired, and 3 were categorized in other professions. The demographic information of the respondents is shown in Table 2.

**Table 2:** Demographic distribution.

Category	Item	Count	Percentage (%)
Biological sex	Female	227	56.33
	Male	176	43.67
Age	18-25	68	16.87
	26-34	196	48.64
	35-50	114	28.29
	Over 50	25	6.2
Education	Bachelor’s Degree	327	81.14
	Below High School	22	5.46
	Master’s Degree or Above	54	13.4
Occupation	Student	58	14.4
	Corporate Employee	134	33.25
	Government Worker	68	16.87
	Technical Professional	64	15.88
	Freelancer	66	16.38
	Retired	5	1.24
	Other	3	0.74

### 4.2. SEM procedure

A SEM was constructed to conceptualize how users form behavioral intentions toward DCCPs and to explore the factors that shape these intentions. Based on the considerations in earlier sections, this study proposes that media richness, design aesthetics, and high culture serve as exogenous variables, whereas perceived cultural value, satisfaction, and cultural identity function as mediating variables; use intention is specified as the endogenous variable. Once the model is specified and the parameter estimates are obtained, it becomes essential to evaluate the model’s adequacy. The analysis and interpretation of SEM typically follow a two-step approach: first, assessing the measurement model; second, examining the structural model [85]. Specifically, the structural model illustrates the hypothesized relationships among latent constructs, while the measurement model captures the linkages between latent variables and their corresponding observed indicators, also referred to as manifest variables [86]. The analytical results based on these evaluations are reported in the subsequent sections.

### 4.3. Measurement model

The suitability of the measurement model was evaluated through CFA, by verifying the reliability and validity of the constructs. In accordance with the guidelines outlined by Fornell and Larcker [87], reliability was assessed using factor loadings, Cronbach’s alpha, and Composite Reliability (CR). The results of the reliability analysis indicated Cronbach’s alpha values ranging from 0.811 to 0.900, exceeding the recommended threshold of 0.70 and thus demonstrating strong internal consistency. Convergent validity was established based on two commonly accepted criteria [86,87]: first, the standardized factor loadings for all items ranged from 0.747 to 0.827; second, the Average Variance Extracted (AVE) values ranged from 0.590 to 0.816, all well above the minimum acceptable level of 0.50 [87]. As shown in Table 3, the results provide strong evidence that the measurement items exhibit adequate reliability and convergent validity.

**Table 3:** Results of construct validity and reliability analysis.

Latent Variable	Measurement Variable	Mean	Std. Dev	Factor Loadings	Cronbach’s alpha	CR	AVE
Media	MR4	3.308	0.972	.786	.859	.786	.797
Richness	MR3			.766			
	MR2			.772			
	MR1			.786			
Design	DA3	3.317	0.998	.776	.820	.790	.814
Aesthetics	DA2			.781			
	DA1			.773			
High	HC4	3.334	1.016	.827	.874	.799	.816
Cultural	HC3			.813			
	HC2			.806			
	HC1			.742			
Perceived	PCV1	3.341	1.002	.786	.868	.783	.780
cultural	PCV2			.797			
value	PCV3			.784			
	PCV4			.788			
Satisfaction	SAT4	3.259	1.047	.790	.880	.790	.766
	SAT3			.814			
	SAT2			.799			
	SAT1			.816			
Cultural	CI6	3.289	0.947	.758	.900	.900	.599
identity	CI5			.764			
	CI4			.783			
	CI3			.780			
	CI2			.761			
	CI1			.797			
Use	UI1	3.355	0.988	.790	.811	.812	.590
intention	UI2			.766			
	UI3			.747			

Discriminant validity was evaluated using the Fornell-Larcker standard [87]. According to this standard, the square root of the AVE for each construct should exceed the highest correlation with any other construct. As shown in Table 4, the square roots of the AVE values for all constructs are greater than the inter-construct correlations, thereby confirming the presence of satisfactory discriminant validity.

**Table 4:** Discriminate validity of the research model.

Constructs	MR	DA	HC	PCV	SAT	CI	UI
MR	.893						
DA	.427^**^	.902					
HC	.384^**^	.384^**^	.903				
PCV	.407^**^	.407^**^	.430^**^	.883			
SAT	.449^**^	.449^**^	.469^**^	.471^**^	.875		
CI	.391^**^	.391^**^	.428^**^	.390^**^	.447^**^	.774	
UI	.338^**^	.338^**^	.415^**^	.415^**^	.374^**^	.373^**^	.768

As presented in Table 5, the results indicate that the overall fit of the model is satisfactory, with all indices meeting the recommended thresholds. Specifically, the chi-square/df ratio was 1.112, falling within the acceptable range of 1.0 to 3.0. The TLI and CFI values were 0.993 and 0.994, respectively, all exceeding the recommended value of 0.90. The AGFI value was 0.827, slightly less than 0.9. Additionally, the RMR was 0.042, which is below the threshold of 0.05, and the RMSEA was 0.017, well under the suggested maximum of 0.08. These results collectively suggest that the model demonstrates a good overall fit.

**Table 5:** Results of construct validity and reliability analysis.

Model	X^2^	X^2^/DF	AGFI	TLI	CFI	NFI	RMSEA
Recommended criteria	p > .05	< 5.0	>.90	>.90	>.90	>.90	<.08
Measurement model	365.755	1.112	0.927	0.993	0.994	0.942	0.017
Research model	627.389	1.845	0.881	0.946	0.952	0.901	0.046

### 4.4. Structural model

**Fig 2:**
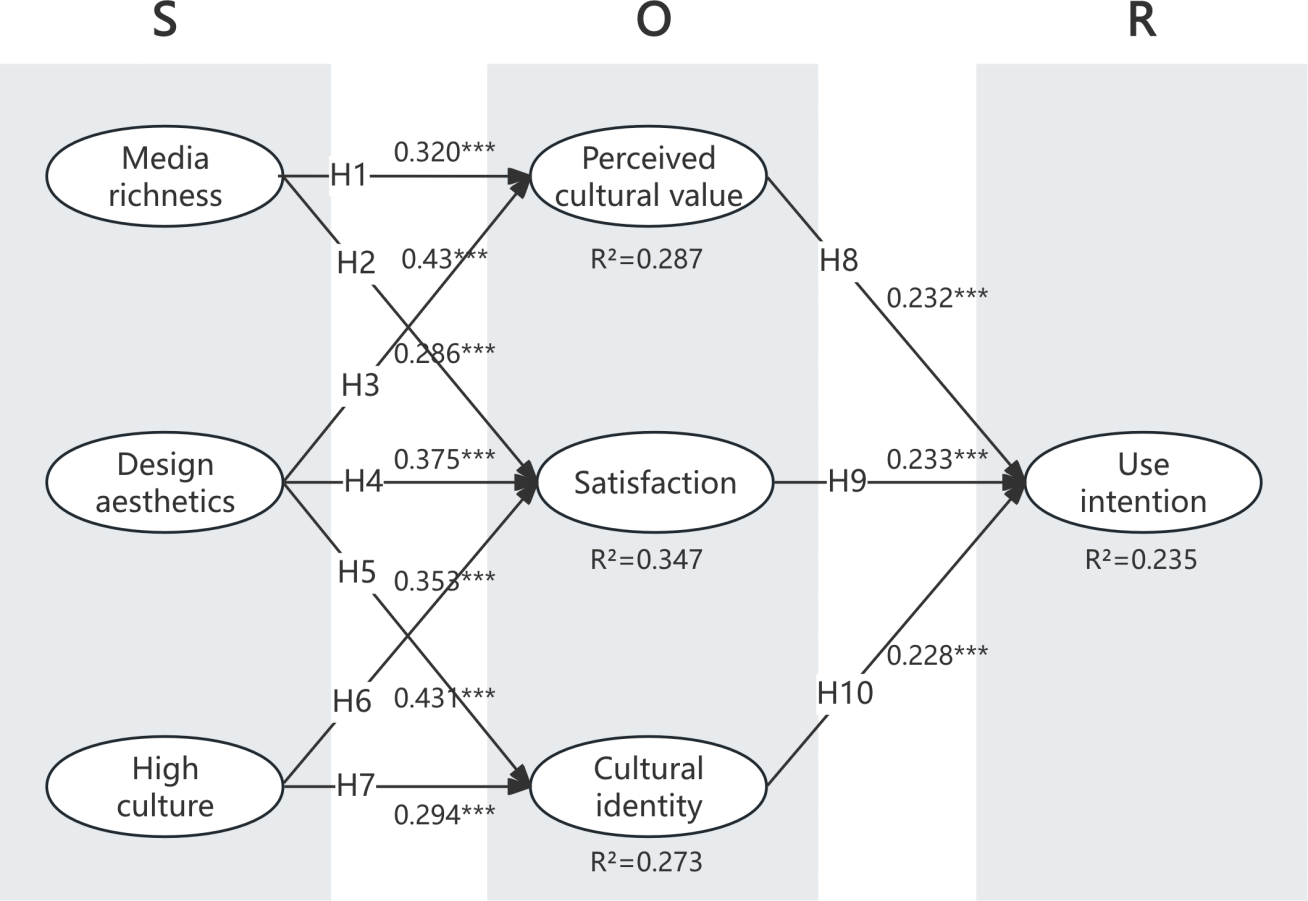
The research model with its standardized coefficients. (A) Bold solid line: research hypotheses opposite to expectations, solid line: supported hypotheses, dashed line: rejected hypotheses. (B) ***p < .001, **p < .01, *p < .05.

The results of the structural model analysis are presented in Fig 2 and Table 6. Initially, the findings indicate that both perceived cultural value (β = 0.058, p < 0.001), satisfaction (β = 0.063, p < 0.001) and cultural identity (β = 0.061, p < 0.001) have notable positive effects on users’ use intention, corroborating H8, H9 and H10. Secondly, media richness is observed to have a notable positive impact on perceived cultural value (β = 0.320, p < 0.001) and satisfaction (β = 0.286, p < 0.001), thus affirming H1 and H2. Thirdly, design aesthetics demonstrates a significant positive effect on both perceived cultural value (β = 0.430, p < 0.001), satisfaction (β = 0.375, p < 0.05), and cultural identity (β = 0.431, p < 0.001), supporting H3, H4, and H5. Lastly, high culture is observed to have a notable positive impact on satisfaction (β = 0.353, p < 0.001) and cultural identity (β = 0.294, p < 0.001), thus affirming H6 and H7.

**Table 6:** The results hypothesis test.

Hypotheses	Hypothesized path	β	S.E.	C.R.	P	Result
H1	MR → PCV	.320	.053	5.819	***	Supported
H2	MR → SAT	.286	.048	5.412	***	Supported
H3	DA → PCV	.430	.058	7.284	***	Supported
H4	DA → SAT	.375	.052	6.671	***	Supported
H5	DA → CI	.431	.054	7.363	***	Supported
H6	HC → SAT	.353	.046	6.609	***	Supported
H7	HC → CI	.294	.045	5.550	***	Supported
H8	PCV → UI	.232	.058	3.908	***	Supported
H9	SAT → UI	.233	.063	3.833	***	Supported
H10	CI → UI	.228	.061	3.884	***	Supported

The study analyzed direct, indirect, and total effects among media richness, design aesthetics, high culture, cultural identity, satisfaction, perceived cultural value, and use intention (Table 7). The findings provide empirical support for H11–H13. Specifically, Perceived cultural value mediates the relationships between media richness (β = 0.320) and design aesthetics (β = 0.430) and use intention, with total indirect effects of 0.141 and 0.286, respectively, supporting H11. Satisfaction mediates the relationships between media richness (β = 0.286), design aesthetics (β = 0.375), and high culture (β = 0.353) and use intention, with corresponding total indirect effects of 0.141, 0.286, and 0.149, supporting H12. Cultural identity mediates the influence of design aesthetics and high culture on use intention, with significant indirect effects of 0.286 and 0.149, respectively, supporting H13.

**Table 7:** Direct, indirect and total effects among the variables.

Dependent variable	Independent variable	Direct effect	Indirect effect	Total effect
CI	HC	0.294		0.294
	DA	0.431		0.431
SAT	HC	0.353		0.353
	DA	0.375		0.375
	MR	0.286		0.286
PCV	DA	0.430		0.430
	MR	0.320		0.320
UI	HC		0.149	0.149
	DA		0.286	0.286
	MR		0.141	0.141
	CI	0.228		0.228
	SAT	0.233		0.233
	PCV	0.232		0.232

## 5. Discussion

This study takes the DCCP the *Forbidden City 365* as a case and explores the formation mechanism of users’ use intention based on the SOR theory. It systematically analyzes the process from stimuli (factors related to DCCPs), through organism (users’ psychological and emotional states), and finally to response (users’ behavioral intention). Based on empirical data collected from 403 users of the *Forbidden City 365* app, this study reveals that users’ use intention with DCCPs results from the interplay of several contributing factors. All proposed hypotheses were validated, highlighting the importance of the variable interactions embedded within the conceptual framework.

The findings show that media richness positively contributes to users’ sense of cultural value and overall satisfaction. This suggests that the use of diverse media forms—such as images, audio, and interactive video— enhances users’ understanding and acceptance of cultural information, thereby improving the overall user experience. These results align with the observations of Li et al. [38], who underscored the importance of media expressiveness in fostering cultural understanding and satisfaction. Furthermore, the analysis reinforces the importance of thoughtful media design in the context of digital cultural dissemination. Enhancing media richness within DCCPs contributes to creating experiences that are more immersive, pleasurable, and interactive, thereby improving the efficiency of cultural communication. For instance, the integration of advanced tools like augmented reality (AR) and virtual reality (VR) allows these products to deliver dynamic, multifaceted, and layered media presentations. These technologies not only intensify visual and auditory stimuli but also enhance user interaction and engagement, making the experience more vivid, enjoyable, and personalized. High media richness significantly promotes users’ emotional resonance and cultural identity deepening their perception of cultural value and contributing to the inheritance and innovation of cultural heritage.

Design aesthetics were also found to have a significant positive impact on perceived cultural value, satisfaction, and cultural identity, thereby further enhancing users’ use intention. As the visual carrier of cultural content, design aesthetics reinforce the intuitive presentation and emotional appeal of cultural information, helping to foster a stronger sense of cultural identity among users. This finding aligns with the perspective of Hagtvedt and Patrick [45], who posited that aesthetic experiences can enhance user satisfaction and behavioral intentions, and it echoes the conclusion by Li & Li [46] that aesthetic appeal can evoke cultural resonance. These results demonstrate that design aesthetics are not only a source of visual pleasure but also a key driving force behind increased satisfaction, cultural identity and perceived cultural value. Cultural institutions can attract and retain users through sophisticated visual design, enhancing the overall aesthetic quality and perceived value of their products. High-quality aesthetics in digital products are reflected not only in color coordination, interface layout, and graphic harmony [39] but also in the modern reinterpretation of traditional cultural symbols [51], conveying cultural meaning through visually striking and emotionally evocative forms. Well-designed interfaces and interactive experiences can elicit aesthetic enjoyment and emotional connections, thereby improving satisfaction and user loyalty. Moreover, the originality and uniqueness of visual design help distinguish a product from its competitors, enhance brand image and cultural influence, and promote the dissemination and preservation of culture in the digital age.

High culture depth was also shown to positively influence both user satisfaction and cultural identity, underscoring the value of cultural richness in fostering users’ sense of pride and belonging. This conclusion is consistent with Kweon [53], who found that digital products with cultural depth can enhance users’ emotional identification, and it supports Li [49], who noted that high culture content significantly improves user satisfaction. Cultural depth does more than just help users feel connected to a product — it also significantly enriches their overall experience. When DCCPs weave in elements that reflect the essence of traditional culture, they open the door to exploring deep-rooted values and symbolic meanings. By thoughtfully incorporating iconic cultural symbols, colors, and patterns, these products not only stand out but also offer content that feels authentic and meaningful.

Furthermore, perceived cultural value, satisfaction, and cultural identity all have a significant positive impact on use intention, indicating that users’ cognitive and emotional experiences serve as key mediating variables in driving behavioral engagement. Specifically, the sense of cultural meaning and value recognition that users derive from DCCPs plays a critical role in encouraging continued usage. This highlights the importance of such products not only to offer visual and formal appeal, but also to convey cultural depth in order to strengthen users’ emotional connection and product stickiness. These findings align with the conclusions of Zheng et al. [71], extending the concept of perceived value into the cultural domain. They underscore how cultural depth and uniqueness can evoke emotional resonance, thereby enhancing users’ willingness to use the product. The positive experiences and psychological satisfaction users gain during interaction with the product form an essential psychological foundation for developing stable usage habits. Satisfaction with the modes of cultural expression, interactive experiences, and aesthetic design significantly boosts users’ use intention. This underscores the role of DCCPs in meeting both emotional and cognitive needs, while also complementing previous research that primarily approached the issue from a technology acceptance perspective [88]. Cultural identity exerts a significant positive influence on use intention, suggesting that the sense of belonging cultivated through interaction with DCCPs fosters emotional resonance, which in turn strengthens user loyalty and engagement [71–73]. When DCCPs successfully elicit users’ identification with local culture, national identity, or shared cultural values, they establish a profound emotional connection that drives long-term participation. This helps foster the ongoing transmission and preservation of cultural values.

The study further reveals that media richness exerts a significant indirect effect on use intention through two mediating variables: perceived cultural value and satisfaction. This finding echoes Zhang et al. [38], who highlighted the contribution of media expressiveness to perceived value and user satisfaction, further reinforcing the importance of media design in the dissemination of digital culture. Design aesthetics demonstrated the strongest indirect effect among the three stimulus variables, suggesting that high-quality visual design not only offers aesthetic pleasure but also deepens users’ sense of identification and belonging with the product’s cultural value. This illustrates the bridging function of design aesthetics in constructing cultural value and fostering emotional connections, in alignment with the conclusions of Jiang et al. [81]. In comparison, high culture influences use intention primarily through satisfaction and cultural identity, emphasizing the critical role of cultural depth in evoking emotional resonance and enhancing cognitive engagement. This supports the findings of Cui [51] and Li [49], who argued that culturally rich content fosters stronger user identification and satisfaction. Overall, design aesthetics emerges as the most direct and influential pathway to stimulating use intention, while media richness and high culture content promote sustained user behavior through multiple mediating mechanisms—namely, perceived cultural value, satisfaction and cultural identity. The multi-path mediation model proposed in this study sheds new light on how users engage with DCCPs. Beyond offering theoretical refinement, it also brings practical value by informing design improvements and strategies to boost user participation. In a broader sense, the results point to meaningful ways of promoting the lasting integration of cultural expression within digital environments.

## 6. Theoretical Implications

This research adopts the SOR framework to explore users’ psychological engagement with DCCPs. Rather than merely applying the model, it develops a context-specific structure that clarifies how users process external factors such as media richness, design aesthetics, and cultural depth. These elements serve as stimuli that shape users’ internal cognition and emotions, ultimately influencing their behavioral intentions. This extends the application of the SOR model into the domain of digital cultural experiences and provides new theoretical insights and empirical evidence for understanding user behavior in DCCPs.

Moreover, the study advances the perceived value theory within the cultural context by refining and emphasizing the construct of perceived cultural value. Unlike conventional studies that focus primarily on functional or economic value, perceived cultural value highlights users’ identification with and emotional resonance toward historical traditions, aesthetic styles, and national symbols. The empirical findings confirm that perceived cultural value has a significant positive effect on users’ behavioral intentions. This reinforces the theoretical argument for integrating cultural elements into technological frameworks, thereby enriching both the behavioral study of digital users and the conceptual development of perceived value theory in cultural domains.

By doing so, the study addresses an important research gap: how external design and cultural stimuli interact with users’ internal psychological mechanisms to shape engagement and intention in digital cultural contexts. It thus contributes to the growing interdisciplinary discourse connecting cultural studies, design aesthetics, and user experience theory.

## 7. Practical implications

This research provides actionable insights for practitioners and policymakers in the digital cultural-creative industry.

First, enhancing media richness through multimodal integration—such as the use of images, sound, and interactive functions—can effectively heighten user immersion and perceived cultural value, thereby improving satisfaction and engagement.

Second, design aesthetics play a crucial role beyond visual appeal; they function as vehicles for cultural communication. Designers should harmonize cultural symbols, artistic styles, and users’ emotional experiences to strengthen both cultural identity and emotional connection.

Third, high culture serves as a core driver of users’ cultural identity and satisfaction. Managers and content developers should integrate deep historical traditions and national symbols into digital products to evoke emotional resonance and convey cultural significance.

Furthermore, the findings highlight perceived cultural value, satisfaction, and cultural identity as key mechanisms sustaining user engagement. Practitioners should therefore emphasize users’ cultural value experiences throughout product design, interface development, and operational strategies.

Ultimately, this research advocates for a transformation in DCCP development—from a function-oriented model to a culture-oriented model. Such a shift not only enhances user loyalty and brand value but also strengthens the effectiveness of digital cultural dissemination and the sustainable growth of the cultural-creative industry.

## 8. Limitations and suggestions for future research

Despite highlighting the dual optimization of cultural communication and user experience in DCCPs, some limitations are inherent in this study. First, the exclusive focus on the *Forbidden City 365* as a single case limits the representativeness of the sample, making it difficult to generalize the findings to other types of digital products. To strengthen external validity, future investigations might explore a wider variety of product types. Second, this study primarily examined product-related features while overlooking user characteristics and sociocultural factors. It is recommended that future research incorporate variables such as cultural motivation, digital literacy, and platform mechanisms to develop a more comprehensive model. Third, future research should further investigate the shift in DCCP development models from a “function-oriented” to a “culture-oriented” approach, in order to verify whether this transformation can indeed enhance user loyalty, strengthen brand value, and improve the effectiveness of cultural dissemination. Finally, as a cross-sectional study, the current research cannot establish causal relationships. Longitudinal or experimental designs are encouraged in future studies to dynamically track changes in user behavior over time.

## Supporting information

S1 Data Raw data. (XLSX)
